# Moderated moderation modelling of subjective social status, pocket money and depressive symptoms of university students in Ghana

**DOI:** 10.3389/fpubh.2024.1325441

**Published:** 2024-04-04

**Authors:** Frank Quansah, Medina Srem-Sai, Edmond Kwesi Agormedah, Francis Ankomah, John Elvis Hagan, Thomas Schack

**Affiliations:** ^1^Department of Educational Foundations, University of Education, Winneba, Ghana; ^2^Department of Health, Physical Education, Recreation and Sports, University of Education, Winneba, Ghana; ^3^Department of Business and Social Sciences Education, University of Cape Coast, Cape Coast, Ghana; ^4^Department of Educational Studies, Patton College of Education, Ohio University, Athens, OH, United States; ^5^Department of Health, Physical Education and Recreation, University of Cape Coast, Cape Coast, Ghana; ^6^Neurocognition and Action-Biomechanics-Research Group, Faculty of Psychology and Sports Science, Bielefeld University, Bielefeld, Germany

**Keywords:** mental health, university students, pocket money, adulthood, social status

## Abstract

**Introduction:**

Although the relationship between subjective social status and depression in university students has been well-established, this association could be seen as a spurious one. Previous studies have shown that key variables like financial resources and age could play key roles in explaining the variances in social status and mental health outcomes. In this research, we assessed the complex interrelationships between subjective social status, financial resources at their disposal and depressive symptoms among university students within their young and middle adulthood stages.

**Methods:**

A cross-sectional survey was conducted in a university in Ghana to sample 1134 university students through accidental sampling. The McArthur Scale and WHO-5 Well-being measure were used for the data collection.

**Results:**

The results revealed that higher levels of subjective social status were associated with lower levels of depression. It was further found that the interaction between students’ pocket money and age played unique roles in the relationship between subjective social status and depression.

**Conclusion:**

The study findings call on stakeholders in education to explore funding opportunities and to examine ways of empowering parents (financially) to adequately support the students. Health educationists and promoters, including psychologists, school counsellors and parents could compliment these efforts by helping to train and empower students through self-regulation or management skills to help improve their well-being. Continuous efforts are required to improve the financial status and mental health of students.

## Introduction

University students worldwide have been recognised as a group at increased risk of experiencing mental health challenges, especially depression, which is considered the leading cause of many disabilities and illnesses globally (1, 2). Depression is a psychological sickness characterised by chronic feelings of sadness, unhappiness, hopelessness, and apathy (2), affecting more than 280 million people worldwide (3). This chronic condition influences people’s discernments, feelings, behaviours, academic life, financial status, and relationships (4, 5) of which college learners are not excluded. Other scholars have reiterated that depressed patients are vulnerable to suicide, infectious diseases, and substance abuse (6–10).

Previous studies among university students have revealed extremely high to moderate depressive symptoms among this population, with a rising prevalence rate (11–17). For example, scholars have discovered that depression among students ranges from 10 to 85%, with reported depression rates being more than what has been identified in the general population (14, 18, 19). Within the African context, depression prevalence rates have been noted among university students in Nigeria (7%), Kenya (25.2%) and Ghana (39.2%) (20–22).

Subjective social status (SSS) is generally considered as a significant predictor of psychological well-being and mental health in various samples, such that people who evaluate themselves as having higher social status or economic well-being in life are likely to feel better and healthier than the those who perceive themselves otherwise (23–27). The SSS of individuals is assessed from the perspective of objective social status. Objectively, social status is estimated based on the amount of resources people possess using three main indicators: income, education, and work/occupation (28). On the other hand, SSS reflects how people (inter-subjectively) assess their status relative to others in society based on income, education, and work (29–31). Unlike the objective social status, the SSS provides individuals the opportunity to judge which indicators of the objective social status are the most important determinants of their SSS (32, 33). This measure provides a sense of inner judgement, satisfaction, and joy because individuals rate how they see themselves and are not necessarily bracketed in any fixed income benchmark. For instance, with a particular income level, one may be rated as belonging to the low social class on the objective scale, however, such individual may be content with that income level and possibly rate themselves subjectively as belonging to the high social class group.

In Africa, and particularly, in Ghana, it has been observed that university students (and even pre-tertiary students) have the tendency to rate themselves on how well they see themselves relative to others (34, 35). Previous studies on the SSS and mental health outcomes have reported that people’s impression of their social status affect their health and overall well-being (24). Interestingly, Hoebel et al. (36) in their research observed that the SSS of individuals had greater chances of affecting mental health compared to the objective measures of social status (socio-economic factors). According to the social comparison theory (SCT), much of this social evaluation is due to people’s efforts in improving their lives (37, 38). A review of 53 previous investigations by Hegar et al. (39) showed that low SSS was associated with different symptoms of illness, including depression, even when controlling for socioeconomic factors such as income, employment, and educational level (36, 40). Among university students, Rubin et al. (41) found a positive connection between subjective view of social status and social connectedness with peers. Rubin et al. (41) also found that SSS and social contact with peers negatively predicted depression but positively predicted well-being, suggesting that higher SSS can reduce depressive symptoms among college students.

Several earlier studies have shown that financial resource is a significant variable that affect the strength and direction of the relationship between SSS and depression among college students (12, 42–54). What is remarkable about previous research is that students who report financial stress and strain are more likely to experience depressive symptoms. In addition, these scholars found that university students often have financial challenges, with those from poor background (less privileged families) exhibiting intense anxiety and depressive symptoms. In a discussion of the reciprocal relationship that exists among societal position (social status), economic resources, and mental health, it has been demonstrated that people’s evaluation of social status can have significant and serious health repercussions beyond the influence of objective socioeconomic status indicators (24, 26, 27, 36, 40, 55).

One of the approaches to assessing the financial resources of university students is through the money they have at their disposal (47). Pocket money (allowance) is a common phenomenon used as an indicator of financial resource availability for students who live away from home and need money for various purposes (56). Research evidence has shown a positive association between the socio-economic background of students and their pocket monies (57). In this research, pocket money was used to depict the financial resources at the disposal of the students, including monies from family relatives, parents/guardians and friends for support (58–62).

Situated within the self-determination theory, it is suggested that having enough money helps students socialise, increase their independence, build relationships with peers, and even demonstrate higher potentials in their academic work (63). Subsequently, the lack of financial support (i.e., inadequate pocket money) affects the satisfaction of these psychological needs and leads to psychological problems (depression) and feelings of negative social status (6, 64). The longstanding relationship between SSS and depression has been found to be a function of age of the individuals in question, especially for university students (5, 12, 65). For instance, Chen et al. (12), for example, found that older students suffer high levels of depression than younger students because they have more stressors emerging from work/employment, marriage, finances/economics, and graduation which potentially affect their SSS. This understanding, therefore, suggests that the connection between SSS and mental health may differ between young and middle-aged students (36).

Ghana is classified as a lower-middle income country, and there is considerable evidence that the level of multi-crime poverty among its entire population (13.6 million, 44.1% to 14.4 million, 46.7%) continues to rise. In addition, the global poverty rate increased from 11.1% in 2019 to 11.3% in 2022 (66–68). In this population, most students have been supported by their parents or guardians to attend school since childhood. Therefore, in light of the above, college students from different socioeconomic backgrounds may experience mental health challenges due to poverty-related problems.

Although depression has been studied globally and to some extent in Ghana, majority of these studies have focused on the assessment of the prevalence of depressive symptoms among different populations in Ghana (20, 50, 69). Additionally, several mental health researchers have focused primarily on investigating objective variables of socio-economic status of people, including educational level, income and employment, and ignored the subjective measure of social status. Further, there is a paucity of evidence about the link between appraisals of SSS and the risk of depression (36). To date, little is known in Ghana considering the unique relationships between SSS and depression, with financial resources (i.e., income or pocket money) and age as moderators in these associations. It is recently that Quansah et al. (47) examined the role of monetary resource in the association between SSS and well-being among adolescents in schools. Although the authors established that monetary resource moderated the link between SSS and well-being, the study was carried out among secondary school students and age was not moderated as well.

**Figure 1:**
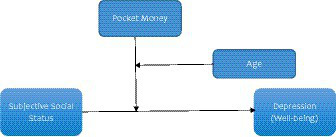
Conceptual framework.

Considering that mental health among university students is getting worse over time, with high rates of mental disorders involving depression (14, 42), there is an urgent need for more information on how SSS, pocket money, age and depression are connected to create further awareness, promote mental health, and minimise depressive disorders among university students. The findings of this study could help provide additional insight to stakeholders to guide mental health policies in higher institutions and interventions aimed at primary prevention and minimization of mental disorders among university students. The rationale of this study was to examine the moderated moderation of SSS (independent variable), and depression (criterion variable), with financial resources and age as moderators among university students in Ghana. Three specific objectives were addressed; (1) assess the relationship between SSS and depressive symptoms of university students, (2) examine the role of pocket money in the relationship between SSS and depressive symptoms of university students and (3) examine whether the relationship between SSS and depression as moderated by pocket money differ across age among Ghanaian university students. The conceptual framework linking the study variables is illustrated in Figure 1.

## Materials and methods

### Design and participants selection

The descriptive cross-sectional survey design was employed to accidentally select the sample for the study. The sample (*n =* 1,134) covered university students from the University of Education, Winneba in Ghana. The sample size for this study was determined using *a priori* statistical software G*Power version 3.1.9.2. The participants were in their 1 to 8 semesters. A semester typically is made up of 14 to 16 weeks of in-school operations. This period encompasses lectures and examinations.

### Measures

Data were gathered using a questionnaire from previously validated scales. The questionnaire contains items on SSS, depression, pocket money and demographic profile of respondents.

#### Predictor: subjective social status

The SSS of the students was measured using McArthur’s Scale (29, 70). SSS is a single-item measure that assesses a person’s perceived rank (social standing) relative to others in a group. In this measure, participants were presented with a ladder with each rung labelled with a number between 1 and 10 with the higher scores suggesting higher SSS. The rungs represent where people stand in relation to others in their community or school. The higher rungs (i.e., the top of the ladder) represent those who are better off—they have more money, education, and better jobs and those at the bottom are the people who have the lowest standing in the community or school—who have the least money, least education, and the least respected jobs or no jobs. Participants are asked “at this time in your life, relative to other people in your community or school, what rung of the ladder do you think you stand on, from 1 (worst off) to 10 (better off)? The McArthur scale is psychometrically valid and reliable in English (29, 70, 71). The single score provided by each participant was used for analyses.

#### Criterion: depression

University students’ depression as a criterion variable was assessed using the WHO-5 well-being index (72, 73). The WHO-5 is conceptualised as a unidimensional measure with each item rated on a six-point Likert scale ranging from 0 (at no time) to 5 (at all the time). Some typical items on the scale include “I have felt active and vigorous,” “I have felt cheerful and in good spirit” and “I woke up feeling fresh and rested.” The raw score theoretically ranges from 0 (lowest well-being) to 25 (highest well-being). For ease of data analysis, the overall score of 25 is multiplied by 4 to obtain a composite score ranging from 0 to 100. The WHO-5 has adequate validity in screening for depression among several cohorts including university students (74–76). Quansah et al. (75) have found the WHO-5 well-being index to be an appropriate instrument for screening depression in Ghana with fair divergent and convergent validity estimates. In screening for depression, lower scores (i.e., <50) suggest depressive symptoms while higher scores (>50) imply sufficient well-being of the participant. Using the McDonald Omega method, the data for this scale in this study yielded a reliability estimate of 0.811.

#### Moderator variables: pocket money and age

In this current investigation, students’ pocket money was operationalised as financial resources or income at their disposal. This may be money for school fees, or money for buying food and school-related materials (59, 61, 62, 77, 78). The respondents were asked to rate the financial resources at their disposal whether they received from their parents/guardians (or other sources) using response options ranging from no money to completely sufficient money; this measurement approach is supported by Chun et al. (79).

The age of the students was also used as moderating variable. The age of the respondents ranged from 18 years to 42 years old. The mean age was 27 years. Participants who were between 18 and 25 years were classified as young adults whereas participants who were within the age range of 26 to 42 were considered to be in their middle adulthood stage. About 46.3% (*n =* 525) of the participants were within the young adulthood stage whereas 53.7% (*n =* 609) were found to be within the middle-adult group. It could be observed that the greater proportion of the sample were middle-adult students which may not reflect a typical university population, especially in most westernised world.

This age-sample distribution is attributed to some factors. First, the rate of poverty in Ghana is on the rise and at the highest point than in previous years (80). This situation has resulted in delayed enrolment into higher education institutions due to financial challenges. A common practice is that most individuals delay their tertiary education after secondary school to work to amass financial resources to be used to further their education. Secondly, a significant number of students from the University of Education, Winneba (where this study was conducted) are people who have diploma degree and seeking for Bachelor’s degree. Such students have gone through 3 years of tertiary education either at the training college or technical university (81). Previous studies conducted in this same university have revealed a similar population distribution in terms of age (82, 83). Besides, the convenient sampling approach adopted could have skewed the sample to include these students with diploma degree qualification. These dynamics resulted in the nature of age distribution of the university students.

#### Covariates

Three demographic variables of students including sex (male vs. female), educational level (bachelor vs. postgraduate) and the number of semesters (1 to 8) were controlled as covariates. Dummy variables were created for these variables. For gender, the female category was used as the reference group. Regarding educational level, bachelor’s degree was used as the reference group and semester 1–2 was used as a reference group for the number of semesters.

#### Data collection procedure

Prior to the data collection, ethical protocols were ensured. The study was approved by the Institutional Review Board of the University of Education, Winneba, Ghana with reference number DAA/P.1/Vol.1/39. The study was conducted among undergraduate students at the University of Education, Winneba in Ghana who had agreed to partake in the study. The data collection took place at the premise of the university. Participants were contacted for their availability and willingness to participate in the study. The data collection commenced from February to March 2021 (2 months). This was the period the schools had begun a new academic year after COVID-19 had subsided. Two research assistants were employed and trained to help collect data for the research. As part of the training, participants were systematically guided through each item of the instrument to help them clearly understand and use the instrument without confusion. The survey instruments were given to the participants immediately before lectures to respond to the survey items within 20 to 25 min with the help of the research assistants. The items on the questionnaire were explained to the respondents to avoid any misinterpretation. Apart from ensuring that all COVID-19 safety protocols have been adhered to, ethical considerations such as anonymity and confidentiality were also maintained. The students were asked not to write their names on the questionnaire, and they were assured that their identity would not be revealed to anybody. Further, they were assured that any data provided would be used solely for academic purposes. The selected students were asked to sign the informed consent form.

### Data analyses strategy

The data analyses started with descriptive and bivariate computations to explore the data and understand the associations existing among the major variables of the study. Using sex, education level and the number of semester as covariates, regression-based moderated moderation analysis was performed to address the research questions. For the moderated moderation analysis, age and pocket money were used as the primary and secondary moderators, respectively. SSS was used as the predictor and depression level was used as the criterion variable. The Hayes PROCESS framework was used to model the relationship existing between the variables. We used 10,000 bootstrap samples using Model 3. Significant results were evaluated on the basis that the confidence interval for the parameter estimate did not include zero. All inferential analyses were performed at 95% confidence level with an alpha of 0.05. SPSS (version 25) computer programme was used to process the data analyses.

## Results

### Descriptive and bivariate analysis

The study comprised more male students (72.3%) compared to females across young adults and middle adulthood stage. Over 80% of the participants read programmes at the Bachelor’s degree level. The students reported a varying number of semesters they have been on campus. All the students reported that they have received some level of pocket money. For instance, a greater proportion of them indicated that the pocket money was not sufficient (31.8%) or less sufficient (49.6%). The perceived social status reported by the participants were from 3 to 5 (with 10 being the highest score suggesting highest SSS and 1 being the least) among students in the young and middle adulthood stage. Depression levels were reported to be low by most of the participants (Table 1).

**Table 1:** Descriptive and bivariate analysis.

Variables	Levels	Young adults (*n* = 525)		Middle adults (*n* = 609)		Overall cases		𝛘^2^ (*p* value)
		n	%	n	%	n	%	
Sex	Male	326	62.1	494	81.1	314	27.7	50.948 (*p < 0*.001)
	Female	199	37.9	115	18.9	820	72.3	
Education level	Bachelor’s degree	448	85.3	496	81.4	944	83.2	3.056 (*p = 0*.080)
	Master’s degree	77	14.7	113	18.6	190	16.8	
Semester	1^st^ – 2^nd^ Semester	85	16.2	20	3.3	105	9.2	78.172 (*p < 0*.001)
	3^rd^ – 4^th^ Semester	326	62.1	489	80.3	815	71.9	
	5^th^ – 6^th^ Semester	104	19.8	75	12.3	179	15.8	
	7^th^ – 8^th^ Semester	10	1.9	25	4.1	35	3.1	
Pocket money	No money	0	0	0	0	0	0	35.602 (*p < 0*.001)
	Not sufficient	156	29.7	205	33.7	361	31.8	
	Less sufficient	234	44.6	329	54.0	563	49.6	
	Sufficient	125	23.8	65	10.7	190	16.8	
	Completely sufficient	10	1.9	10	1.6	20	1.8	
Social status ladder (1–10)	1	5	1.0	0	0	5	0.4	66.206 (*p < 0*.001)
	2	30	5.7	5	0.8	35	3.1	
	3	159	30.3	129	21.2	288	25.4	
	4	195	37.1	235	38.6	430	37.9	
	5	82	15.6	160	26.3	242	21.3	
	6	25	4.8	60	9.9	85	7.5	
	7	10	1.9	10	1.6	20	1.8	
	8	10	1.9	5	0.8	15	1.3	
	9	9	1.7	5	0.8	14	1.2	
	10	0	0	0	0	0	0	
Depressive symptoms	Low (>70–100)	267	50.9	375	61.6	642	56.6	31.442 (*p < 0*.001)
	Moderate (>40–70)	161	30.7	187	30.7	348	30.7	
	High (0–40)	97	18.5	47	7.7	144	12.7	

### Relationship between SSS and depression of university students in the young and middle adulthood stage

First, we examined the connection between SSS and depression among university students, while controlling for some key variables in this study. The analysis revealed that SSS significantly predicted depression in university students in the young and middle adulthood stage, *B =* 10.970, *SE = 0*.815, Boot*CI* (9.371, 12.568; see Table 2). More explicitly, higher levels of SSS were associated with lower levels of depression whiles lower levels of SSS were linked to higher levels of depression.

**Table 2:** Parameter estimates for the relationship between SSS, pocket money, age and depression.

	Coeff	Se	t	LLCI	ULCI
Constant	20.882	6.088	3.430	8.937	32.826
SSS	10.970	0.815	13.465	9.371	12.568
W1	0.264	4.426	0.060	−8.420	8.948
W2	13.639	5.909	2.308	2.046	25.233
W3	21.934	2.921	7.509	17.832	26.964
Int_1	−0.392	1.022	−0.384	−2.397	1.613
Int_2	4.205	1.281	3.283	6.717	10.692
Int_3	5.618	6.140	0.915	6.429	17.666
Z1	−1.730	4.127	−0.419	−9.827	6.367
Int_4	0.909	0.934	0.974	−0.922	2.741
Int_5	0.213	10.136	0.021	−19.675	20.102
Int_6	53.192	12.378	4.297	28.906	77.479
Int_7	−199.575	68.503	−2.913	−333.984	−65.166
Int_8	0.144	2.473	0.058	−4.709	4.997
Int_9	−10.231	2.758	−3.710	−15.643	−4.820
Int_10	55.192	16.861	3.273	22.110	88.274
Sex	4.376	1.348	3.247	1.731	7.020
Education level	−1.174	1.568	−0.749	−4.252	1.903
Semester	0.111	0.441	0.251	−0.755	0.976

Model Summary: R^2^ = 0.347; MSE = 365.423; F(12, 1,121) = 49.537, *p* < 0.001. Test(s) of highest order unconditional interaction(s): X*W–R^2^∆ = 0.080, F(3, 1,121) = 4.311, *p* = 0.005; X*Z–R^2^∆ = 0.001, F(3, 1,121) = 0.949, *p* = 0.330; X*W*Z–R^2^∆ = 0.0160, F(3, 1,121) = 9.657, *p* < 0.001. Age: Z1, Middle Adult, reference group, young adult. Pocket Money: W1, Less sufficient, W2, sufficient, W3, completely sufficient, reference group, Not sufficient. Product terms key (Interactions): Int_1: SSS * W1; Int_2: SSS * W2; Int_3: SSS * W3; Int_4: SSS * Z1; Int_5: W1 * Z1; Int_6: W2 * Z1; Int_7: W3 * Z1; Int_8: SSS * W1 x Z1; Int_9: SSS * W2 x Z1; Int_10: SSS * W3 x Z1.

### Moderating role of pocket money in the relation between SSS and depression of the university students

The study also assessed whether the relationship between SSS and depression in university students differed based on the sufficiency of their pocket money. A significant moderation effect of pocket money was observed in the relationship between SSS and depression, *F*(3, 1,121) = 4.311, *p = 0*.005. Particularly, the moderator, pocket money, contributed about 8% of the variance in depression when all other variables are controlled for. Higher SSS levels were strongly linked to lower depressive symptoms (improved well-being) for students with sufficient (*B =* 4.205, *SE =* 1.281, Boot*CI*[6.717, 10.692]) and completely sufficient pocket money (*B =* 5.618, *SE =* 6.140, Boot*CI*[6.429, 17.666]). The result implies that for students with sufficient pocket money (compared with insufficient pocket money), their SSS could easily enhance their well-being and subsequently, reduce depressive symptoms.

### Moderating role of age in the moderating effect of pocket money in the relationship between SSS and depression among university students

**Figure 2:**
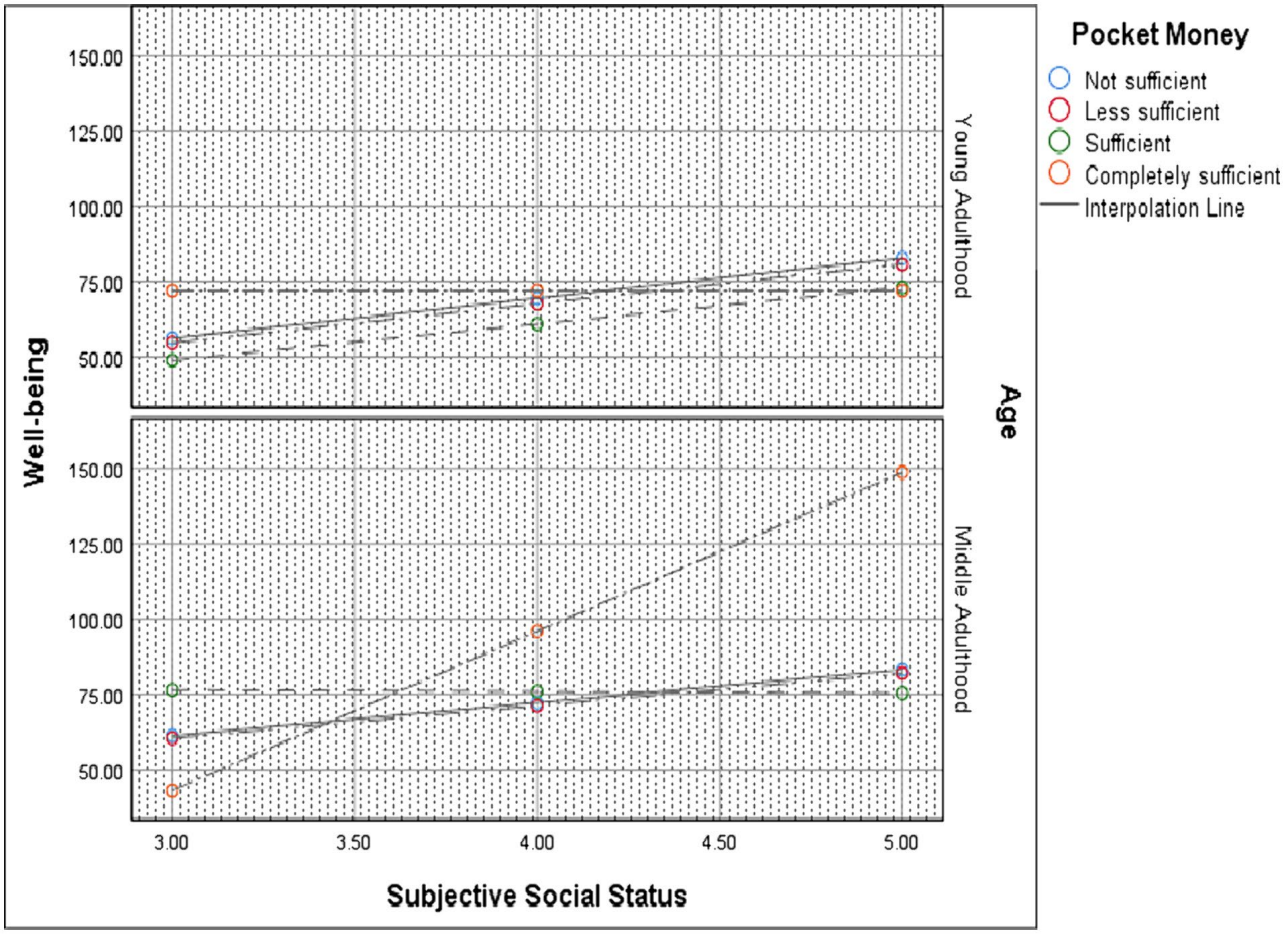
Probing the moderated moderation effects.

We also evaluated the intervening role of the interaction between pocket money and age in the relationship between SSS and depression among university students. It was discovered that age solely failed to moderate the SSS-depression relationship, *F*(3, 1,121) = 0.949, *p = 0*.330. That is, the relationship was not different for students in the young and middle adulthood stages. However, when age interacted with pocket money (acting as moderator), some differences in the SSS-depression relationship were discovered, *F*(3, 1,121) = 9.657, *p < 0*.001. The probing results, as presented in Figure 2, revealed that high SSS levels were strongly associated with low depressive symptoms (improved well-being) for middle-adult students with completely sufficient pocket money (compared to young adults with completely sufficient pocket money).

## Discussion

Although previous research has demonstrated a significant relationship between SSS and depression among university students globally (84–89), the moderating roles of pocket money and age in this relationship have not been the focus of attention previously in the university context. The main contribution of this present inquiry is reflected in the analysis of moderated moderation effect of pocket money and age in the links between SSS and depression in young-adult and middle-adult university students in Ghana.

The study discovered that higher levels of SSS were associated with lower levels of depression whiles lower levels of SSS were linked to higher levels of depression. This finding extends the outcome from earlier researchers who reported that SSS negatively predict depression among university students (84, 88). High SSS among university students is an indication of their perceived predictability and controllability of their academic and social environment (90, 91). Students having high SSS may mitigate the experience of social vulnerabilities because they possess the innate ability to mobilise social support. However, university students with lower SSS are more susceptible to mental and physical health problems (92–94). Our finding is supported by prior investigations that have shown that lower SSS is related to poor mental health among students (85–87, 89, 95, 96).

A slightly different view was held by Collins and Goodman (97) that although high SSS predicted minimal depression, the association is likely to be overblown in cross-sectional research, especially when some important factors are not controlled (e.g., baseline health, age, cognitive impairment, etc). Indeed, we share in the findings of Collins and colleague’s and reiterates these factors when not controlled could influence the students’ own judgement of their human, social and cultural capital. A notable determining factor of SSS is the self-esteem of the participants—this is likely to make a difference in the findings across studies (98, 99). For example, study participants with high self-esteem are more likely to provide higher SSS compared to those with low self-esteem. From the perspective of social comparison process theory, students who make comparisons in the school or society would be happier if they are better off than their group of comparison (37), as this would improve their learning efficacy, self-confidence, and self-esteem. The impact of SSS on depression may have implications for academic achievement because students with higher SSS are less emotionally distressed. The results of this study point to a need to consider SSS in both mental health counselling and academic advising of university students as it may be amenable to intervention.

A positive and significant moderation effect of pocket money in the relationship between SSS and depression (when moderated by age) among university students was identified. For students with sufficient and completely sufficient pocket money, low SSS levels significantly predicted declining well-being and increased depression (when age intervened) and vice versa. These findings are novel in the context of Ghana and Africa, and it lends support to previous studies that have established that students’ monetary resources (i.e., money) mediated the positive association between SSS and mental health problems among students (36, 87). Students who reported having a lower SSS rated lack of money as having a relatively large impact on their mental health, hence, the likelihood of poorer mental health (87). Lower objective SES (as measured by student income) and lower SSS were independently associated with declining psychological health outcomes among students (e.g., depressive symptoms) (36, 46, 47). This finding stresses the significant role of students’ pocket money or income in strengthening the positive association between SSS and mental health outcomes, when age is controlled. This result is not surprising since money is a resource that can be used to buffer the impact of stressful events on mental health. For example, students with sufficient pocket money (i.e., income or financial resources) purchase anything of their choice which may place them on higher status relative to their peers who cannot afford to purchase similar items. Importantly, the students with sufficient pocket money might feel in control of financial strain as a potential stressor in their lives both in school and/ or perhaps at home or elsewhere. Students with more money can afford to pay for stress-relieving activities, such as social activities. The presence of sufficient pocket money among the students might build their academic confidence and resilience (87, 100).

Generally, the university students reported minimal levels of pocket money (i.e., not sufficient, and less sufficient) and those with completely sufficient pocket money experienced low depression compared with their counterparts with sufficient pocket money and less sufficient pocket money. This finding corroborates with reported studies which revealed that students with insufficient pocket money tend to exhibit psychosocial dysfunction and poor mental health problems (56, 101, 102). It must be mentioned that students’ pocket money (i.e., financial resources or income) is considered as social economic status. Accordingly, both students’ social stratification (i.e., SSS) and financial resources or income (pocket money) contribute to socio-economic health. This linkage may act as a buffer in reducing mental health problems (e.g., depression). It suggests that when parents or guardians provide no pocket money or less sufficient pocket money for their wards with the presence of low SSS, such students are likely to exhibit some level of depressive symptoms (89). This finding highlights the essence of a high level of SSS with high sufficient pocket money in ensuring lower levels of depression.

University students with sufficient financial resources (i.e., pocket money) are likely to come from families with high socio-economic status or high-income levels as has been revealed in previous research (78). This observation is quite understandable since the students’ income or financial resources (i.e., pocket money) usually come from the incomes from family relatives (e.g., father, mother, siblings) and thus, a reflection of a higher socio-economic status of the family. Moreover, parenting style plays a crucial role in determining pocket money of university students, regardless of whether the family is wealthy or not (103, 104). In this case, the reported pocket money level by the students may not necessarily be a reflection of their parents’ socio-economic status, as some parents will provide insufficient pocket money in order to regulate their wards spending behaviours.

The findings from this study can be explained from the perspective of fundamental cause theory, which posits that people with poor socio-economic status would have limited access to health, health-related information, and psychological services, which leads to poor mental health (105). Consequently, the poor socio-economic status of students can be equated to less sufficient or not sufficient pocket money leading to depression. This result underscores the tendency of insufficient pocket money resulting in depression among students. University students from lower socioeconomic backgrounds tend to have poorer mental health than students from higher socio-economic status backgrounds (14, 15, 106–109). Low pocket money (i.e., monetary resources or income) among students could lead to financial distress, mental health problems (i.e., depression) and poor academic achievement. This implication supports the findings of previous studies that low or inadequate pocket money was associated with students’ hunger, school dropout, truancy behaviours, late attendance to class, and poor attention span in class (110). University students believed that their mental health suffered because of insufficient income or financial resources (i.e., pocket money) (102, 111–116). As students have indicated their pocket money was insufficient, they are more likely to be vulnerable to psychological and emotional consequences. Consequently, students need pocket money for their personal upkeep and procurement of educational-related materials and other consumables they may need to facilitate their stay. Acquisition of these items may ease the discomfort they are likely to experience in the absence of those materials.

In addressing research objective two, it was discovered that the age of the students failed to moderate the SSS-depression relationship. These findings imply that the significant association between SSS and depression among students is not sensitive to the age of the students. Thus, both young and middle adults have the same level of SSS and depression. This result is consistent with prior research that has shown that the age of students does not moderate the relationship between SSS and depression (5, 65). Our finding that age does not act as a mechanism or buffer in the relationship between SSS and depression among students provides no sufficient support to previous investigations that age plays a vital role in the relationship between SSS and depression among university students (5, 12, 65). Previous studies claimed that older students face more stressors regarding employment, marriage, economic and graduation pressures which could potentially affect their SSS. Conversely, evidence from our data revealed that there is the possibility that holding age constant, a higher SSS among students might minimise depression and improve mental health among young and middle (36). The disparities between the current investigation and the previous studies may be attributed to study contexts, measurement of variables and sample characteristics. The present study was executed in Ghana while the previous studies were carried out in Europe, America, and Asian countries.

Despite this finding, age interacted with pocket money (acting as moderator) to moderate the relationship between SSS and depression. Compared to the young adults with completely sufficient pocket money, high SSS levels strongly predicted declining depression levels for middle-adult students with completely sufficient pocket money. In other words, students in their middle adulthood stage experienced less depression when they report their pocket money to be completely sufficient with the same level of SSS. This finding implies that middle-adult students with sufficient pocket money (i.e., income or financial resources) are unlikely to experience financial difficulties or distress as compared to young adults. As explained earlier, with sufficient pocket money, middle adults can purchase anything of their choice and can afford to pay for stress-relieving activities, such as social activities. This could increase their satisfaction and reduce depression that would arise from financial difficulties.

Drawing on the self-determination theory, having sufficient money could assist students to get involved in social activities to enhance their sense of independence, connect socially with friends, and even feel competent in their academic work (6, 63, 64). From the social comparison theory, middle adults having sufficient pocket money, may rank themselves higher compared to young adults in relation to other students in the school or communities. This could build their sense of belonging and self-esteem leading to higher psychological well-being or reducing mental health problems such as depression. This finding suggests we strengthen the financial resources (i.e., pocket money) of both young and middle adults in school since pocket money is considered an objective socio-economic status to boost their SSS and reduce depression.

## Strengths and limitations

This research draws its strength through the application of a more robust statistical procedure in examining the moderated moderation effect of pocket money and age in the links between SSS and depression in young and middle-adult university students in Ghana. This is a novel study in Ghana because, to the best of our knowledge, the moderated moderation effect of pocket money and age in the linkage between SSS and depression in young and middle adults has not been studied using data from Ghana. The study also relies on the sample size and sample design for its strength. As data were collected using self-administered questionnaires, social desirability bias should have been low, because this type of bias mainly occurs when interviewers are involved in the data collection process. The metric scales of the pocket money and SSS measures and the use of their standardised values in the regression models enabled adequate comparison of their associations with depressive symptoms. The research findings provide solid support to the theory of social comparison process, fundamental cause, and self-determination theory to underscore that the relation between SSS, pocket money and depression is not a straightforward one; but requires other intervening variables.

The present research used a cross-sectional design. Hence, no firm conclusions can be made regarding the causal direction of the associations among variables. Therefore, longitudinal, and intervention studies would provide a much better view of the causal link among the variables. Adopting the self-report measures may affect the validity of responses when recalled responses are not accurate and this may introduce some subjectivity in the responses. There could be also several competing factors in determining pocket money and SSS. Thus, the measurement of pocket money and SSS were not objectively determined, but rather in a subjective manner (i.e., reported by the university students). The subjective measurement of SSS and pocket money, to some extent, depends on cultural values, prior experiences, and interactions with the surrounding environment (117). Future researchers could make use of objective measures of SSS and pocket money. Additionally, the use of convenient sampling may pose a limitation on the representativeness of the sample used in this study (118).

## Practical implications

The study finding demonstrates that among students with poorer financial health, the influence of SSS on mental health is greater. Therefore, future mental health interventions targeting students, especially targeting students with poorer financial health, should incorporate components of strengthening SSS. The study calls on several stakeholders in education and health promotion including psychologists, school counsellors and parents to be involved in the training and empowering process to facilitate superior well-being among university students. Providing financial support and satisfaction, health, and well-being of students should be of high priority among educators and parents. Interventions (e.g., bursaries, grants, loans) should be provided to increase the financial resources or income of young and middle adults in schools. More importantly, parents should be empowered through the creation of jobs so that sufficient levels of pocket money can be provided to university students to help reduce education-related inequities. The university should empower students to mobilise and reflect on the limited resources available to them. Empowering young and middle university students might offer a state of balance for them by reflecting on how to use existing resources to attain better well-being.

## Conclusion

The findings from the current investigation underscore the need for strengthening both the SSS and socio-economic status of both young and middle university students in reducing mental health problems (i.e., depression). Low SSS, insufficient and less pocket money (i.e., financial resources or income) are associated with depression levels in young and middle-adult students. Interestingly, students’ money at their disposal and their age are largely relevant variables in this process. Similarly low to moderate SSS with less sufficient to sufficient pocket money results in worse depression, reflecting the issue of how inequalities generate further inequalities. Planned interventions should focus on students’ pocket money (i.e., financial resources or income) to address social class differences to help promote their mental health, especially on the risk of depression.

## Data availability statement

The raw data supporting the conclusions of this article will be made available by the authors, without undue restriction.

## Ethics statement

The studies involving humans were approved by the Institutional Review Board of the University of Education, Winneba, Ghana with reference number DAA/P.1/Vol.1/39. The studies were conducted in accordance with the local legislation and institutional requirements. The participants provided their written informed consent to participate in this study.

## Author contributions

FQ: Conceptualization, Data curation, Formal analysis, Investigation, Methodology, Software, Validation, Visualization, Writing – original draft, Writing – review & editing. MS-S: Conceptualization, Investigation, Methodology, Validation, Visualization, Writing – original draft, Writing – review & editing. EA: Conceptualization, Methodology, Validation, Visualization, Writing – original draft, Writing – review & editing. FA: Conceptualization, Methodology, Validation, Visualization, Writing – original draft, Writing – review & editing. JH: Conceptualization, Funding acquisition, Investigation, Methodology, Project administration, Resources, Supervision, Validation, Visualization, Writing – original draft, Writing – review & editing. TS: Conceptualization, Funding acquisition, Investigation, Methodology, Project administration, Resources, Validation, Visualization, Writing – review & editing.
